# End-to-end donor screening and manufacturing controls: complementary quality-based strategies to minimize patient risk for donor-derived microbiome therapeutics

**DOI:** 10.1080/19490976.2024.2402550

**Published:** 2024-09-18

**Authors:** Jason Goldsmith, Sarah Tomkovich, John G. Auniņš, Barbara H. McGovern, Jennifer C. Mahoney, Brooke R. Hasson, Christopher W J McChalicher, David S. Ege

**Affiliations:** Product Development, Quality, and Supply (PDQS), Seres Therapeutics, Inc., Cambridge, MA, USA

**Keywords:** Microbiome, microbiome therapeutics, live biotherapeutic products, stool donors, fecal transplant

## Abstract

Advances in microbiome therapeutics have been motivated by a deeper understanding of the role that the gastrointestinal microbiome plays in human health and disease. The FDA approval of two stool-derived live biotherapeutic products (LBPs), REBYOTA® 150 mL enema (fecal microbiota, live-jslm; formerly RBX2660) and VOWST® oral capsules (fecal microbiota spores, live-brpk; formerly SER-109), for the prevention of recurrent CDI in adults following antibiotic treatment for recurrent CDI provides promise and insights for the development of LBPs for other diseases associated with microbiome dysfunction. Donor-derived products carry risk of disease transmission that must be mitigated through a robust donor screening program and downstream manufacturing controls. Most published recommendations for donor screening practices are prescriptive and do not include a systematic, risk-based approach for donor stool-derived products. A general framework for an end-to-end donor screening program is needed using risk management strategies for donor-derived microbiome therapeutic using a matrixed approach, combining the elements of donor screening with manufacturing controls that are designed to minimize risk to patients. A donor screening paradigm that incorporates medical history, physical examination, laboratory testing, and donor sample inspection are only the first steps in reducing risk of transmission of infectious agents. Manufacturing controls are the cornerstone of risk mitigation when screening unwittingly fails. Failure Mode and Effects Analysis (FMEA) can be used as a tool to assess for residual risk that requires further donor or manufacturing controls. Together, a well-reasoned donor program and manufacturing controls are complementary strategies that must be revisited and reexamined frequently with constant vigilance to mitigate risk to patients. In the spirit of full disclosure and informed consent, physicians should discuss any limitations in the donor screening and manufacturing processes with their patients prior to treatment with microbiome-based therapeutics.

## Introduction

Interest in microbiome therapeutics has intensified over the past decade due to greater understanding of the potential role of the gastrointestinal microbiome in health and disease. It is generally recognized that a variety of diseases are associated with compositional and functional disruption of the gastrointestinal microbiome, such as low taxonomic diversity and loss of key species and metabolites. Several lines of research suggest that donor-derived live biotherapeutic products (LBPs) act directly in the gastrointestinal (GI) tract to increase microbial diversity and function and improve clinical outcomes in diseases thought to be due to microbiome disruption.^1^

Initial evidence supporting the development of LBPs came from studies of investigational fecal microbiota transplant (FMT), the transfer of minimally processed human feces from donor to recipient. Investigational FMT was introduced in clinical practice to prevent the recurrence of *Clostridioides difficile* infection (CDI), a common debilitating diarrheal disease characterized by a low diversity microbiome.^2,3^ FMT has also been explored for other diseases thought due to microbiome disruption. However, there are predictable risks associated with FMT, such as the transmission of undetected or untested pathogens shed in stool, which have led to hospitalization and several FDA safety alerts.^4–6^ Minimally processed stool transmits not only bacteria, but presumably also transmits fungi, parasites, viruses, and archaebacteria^7^ that are intrinsic components of the GI microbiome with unknown effects on patients. Currently, there are no regulatory guidelines from FDA or European Medicines Agency (EMA) on donor and donor stool screening for FMT preparation, in contrast with other donor-derived biologicals (eg, EMA urine monograph, blood), leading to trial or product specific practices with resulting variable risk of transmission of known and unknown pathogens.^8^ Several publications have outlined best practices for screening donors for FMT.^9–11^ However, none of these guidelines include an approach toward managing risk for donor stool-derived products that takes into account differing product compositions, manufacturing processes, delivery modalities, and treatment indication as factors influencing screening recommendations.

Two donor-derived LBPs, REBYOTA® (fecal microbiota, live-jslm; formerly RBX2660) and VOWST® (fecal microbiota spores, live-brpk; formerly SER-109) were recently approved by the FDA for the prevention of recurrent CDI in adults following antibiotic treatment for recurrent CDI. The composition, product manufacturing, and delivery modality differ between these two drug products. REBYOTA® is a manufactured FMT product from healthy donors with minimal processing consisting of stool suspension in a saline/PEG solution, filtration, aliquoting and freezing in administration bags for enema delivery.^12^ VOWST® is a purified bacterial spore suspension sourced from healthy donors and composed of predominantly Firmicutes spores, dosed at a specific spore titer with strict limits on non-product bioburden (including, but not limited to, an absence of *E*. *coli*), and provided as an oral capsule formulation.

With the approval of LBPs for the prevention of recurrent CDI, other indications for donor-derived microbiome therapeutics are being explored. However, most published recommendations for donor screening practices are prescriptive and do not take into account: (a) the shifting incidence and prevalence of disease, (b) new emerging pathogens,^4–6^ (c) how to assess risk based on the infection of concern, (d) the availability of validated screening tests, (e) the morbidity associated with infection and (f) the availability of efficacious treatment, should transmission occur. Emerging research on microbiome-linked chronic diseases^13,14^ also emphasizes the need for adaptive donor screening paradigms. Other research applications of stool-derived products from different types of donors, or for use in different patient groups also requires differential consideration of risk and potential benefit.

In contrast to unified donor criteria for blood/plasma/tissue with published guidance, regulators have thus far taken an approach of reviewing each product as a separate therapeutic entity, with a holistic approach to donor criteria and testing that considers differences in product manufacturing and composition. Accordingly, a risk-based approach that takes into account these differences is required to set donor screening paradigms to ensure patient safety. In this review, we provide a general framework for devising a holistic donor screening program using risk management strategies for donor-derived microbiome therapeutics. This framework takes a matrixed approach, combining the elements of donor screening with manufacturing controls that are designed to minimize potential adventitious agents (ie, possibly infectious agents), as well as testing of final drug substance for any residual unwanted components. We also describe examples where differential screening may be warranted.

This approach treats donor screening as a process that is in constant evolution and requires understanding the

- Elements of donor screening and potential pitfalls of laboratory testing
- Latest evidence on adventitious/infectious agents of concern for stool-derived products
- Latest evidence regarding chronic medical conditions that could be transmitted by the microbiome
- Effect of manufacturing processes on reducing the risk of adventitious agents
- Feasibility of testing drug substance/product before release

This type of risk-based approach should be the cornerstone of all donor-based microbiome therapeutics to mitigate transmission risks to patients. As a result, this paper is not designed to provide a prescriptive set of donor screening recommendations that all donor-based microbiome therapeutics adopt, but rather establish a foundational tool that can be adapted to the particulars of a specific therapeutic and periodically reapplied with advancements in scientific evidence, improvement in laboratory testing technologies, or emergence of new infectious agents.

## Elements of donor screening

Appropriate screening of donors includes the following four components: (1) health history determined via questionnaire; (2) physical exam; (3) visual inspection of donations; and (4) laboratory testing, described in detail below.

### Health history

The health history accomplishes several objectives to mitigate risk and should be updated with every donor visit. First, the past and current medical history are used to screen for symptoms suggestive of acute or chronic illness that would disqualify the donor. Screening for symptoms that suggest an acute infection are particularly important since titers of infectious agents are typically highest during this early time period. Additionally, donors are screened for high-risk behaviors, such as substance misuse, high risk sexual history and tattoos from unlicensed facilities, which raise the risk of communicable diseases, such as HIV infection. Any history of chronic illnesses that may be associated with gut microbiome disruption (eg, inflammatory bowel disease) is a key component of medical screening.

A history of international travel has unique considerations for stool donations,^15^ given the variety of enteric pathogens that exist across the world and the inability to test for every possible infectious agent that may have been acquired. Thus, donors should be deferred based on the window of asymptomatic infection for the etiologic agents of concern, with a consideration for the detectability by any implemented testing in the donor (as described in Table 1). This requires a thorough country-by-country review, utilizing up-to-date resources such as WHO and CDC travel advisories, to determine which adventitious agents are present in each country. As testing is often not readily available for pathogens less common in the United States, the full length of the asymptomatic period for the endemic microbe of concern with the longest asymptomatic period in a given country ends up being the deferral period for travel to that country.

**Table 1.** Components of an adventitious agents FMEA.

Category	Risk factor
Probability of Occurrence	Incidence and prevalence of asymptomatic infection
	Level of asymptomatic shedding
	Delivery route-specific transmissibility of agent
	Infectious dose by delivery route
Severity (Virulence)	Severity of potential infection by recipient group
	Ease and availability of treatment should infection occur
Detectability	Detectability by donor questioning and physical exam
	Detectability by laboratory screening in relation to infectious dose
	Timing of detectability in relation to testing schedule and quarantine period*
Mitigation via Manufacturing Processes	Inactivation steps
	Clearance steps
	Detectability via product release testing, relative to infectious dose

*Testing schedule and quarantine time should be established to maximize detectability.

#### Physical exam

In some donation schemes, donors are financially compensated. In these cases, the risk of deception by donors can be higher and, physical examination may serve as a secondary control to verify and assess the accuracy of the self-reported health history. Thorough physical exams can also detect any signs of underlying conditions that the donor is unaware of, such as a rash suggestive of systemic disease (eg, pyoderma gangrenosum and inflammatory bowel disease) or obesity (determined through height and weight measurements to get a body mass index).

### Visual inspection of stool

A critical screening tool is the visual inspection of stool donations, which ensures that the donor does not have active diarrhea, which could indicate underlying infection or microbiome disruption. The Bristol Stool Scale^16^ uses a grading system from 1 to 7 to describe the shape and consistency of stool where 4 is normal, and 6 or 7 indicate loose stool or watery diarrhea, respectively. Visual inspection also allows for assessment of visible blood or mucus, which could be a sign of infection or an underlying medical condition. Thus, visual inspection serves as an important pretest that can identify and eliminate symptomatic individuals with high Bristol Stool Scale scores and/or bloody stools prior to the adventitious agent’s assessment and laboratory screening.

### Laboratory testing

Screening donors via laboratory testing is a key requirement of donor screening, as it can detect pathogen carriage or other medical conditions. However, laboratory testing also has inherent limitations such as the limit of detection of the particular assay and the matrix being tested (eg, blood vs stool, which has many inhibitory substances that add complexity to testing). It is also important to be mindful that laboratory performance is strongly impacted by the pretest probability of infection in the target population being tested. Furthermore, most assays have been developed for diagnostic purposes for symptomatic patients rather than for screening of asymptomatic individuals. These divergent goals influence performance parameters, such as acceptable limits for false negative test results. Finally, misuse of a diagnostic assay rather than a screening assay can lead to clearance of an infected donor and transmission of the missed pathogen to FMT recipients, as reported in 2020.^15^

Furthermore, for infectious agents there is a time window period during which a person can be infected and contagious yet will test negative due to assay performance and/or technical limitations, leading to a false result.^15^ This problem can be mitigated by repeat testing over time, although this approach has its own drawback of increasing the risk of false positive test results. In a donor screening paradigm, false positives are preferred over false negatives, but the increased positivity rate results in a larger donor population to compensate for the deferrals. Tests specifically designed for screening asymptomatic individuals such as for bloodborne pathogens, are calibrated to minimize both false positives and false negatives in a population with a low pretest probability of infection (eg, blood donors). However, stool testing typically uses diagnostic assays whose laboratory performance is designed to inform clinical decision-making in symptomatic patients with a high pretest probability of infection. In this scenario, diagnostic assays are usually calibrated to limit false negatives at the expense of false positives to better inform clinical care, because these assays are designed and used in the setting of clinical care with ongoing symptoms for scenarios with a higher pretest probability for infection. Similarly, laboratory assays can be used to detect acute or chronic medical conditions, but interpretation of out-of-range results may need clinical context. For example, a fasting LDL cholesterol value between 100 and 159 mg/dL may be reported as abnormal since this test result may indicate the need for statin therapy in an individual at risk for heart disease. In contrast, in a donor candidate without any relevant medical or family history, the same result would not have the same clinical implications.

## Assessing adventitious/infectious agents in stool-derived products using failure modes and effects analysis

Infectious agents that are inadvertently introduced into a process or product, such as cell cultures, are referred to as adventitious or extraneous agents. Potential introduction of adventitious agents is a critical concern for all drug products and contamination events have led to serious clinical consequences.^17^ Thus, the pharmaceutical industry and their regulators have set strict standards for sterility or allowable bioburden based on the route of administration.

In the pharmaceutical space, donor-derived products are of particular concern for adventitious agents, as they carry an inherent risk of intrinsic contamination with adventitious agents,^8^ in addition to the potential for contamination with extrinsic agents In the case of blood products, the level of risk varies based on the medical history and lifestyle factors of the donor. However, unlike blood, there is significant intrinsic bioburden in stool, and many organisms carry some level of known and unknown infectious risk. Thus, a rigorous approach is needed to assess the risk of adventitious agents in stool-derived products.

Failure Modes and Effects Analysis (FMEA) are standard industry tools that identify and prioritize risks and how to mitigate them and are recommended to address donor-derived product risks. Translating an FMEA to stool-derived product adventitious agent risk should include the multiple categories of risk factors (Table 1). An FMEA gives a numerical assignment to each factor, and when considered in aggregate allows for identification of, and focus on, the most significant aspects of risk. An FMEA is a living tool. Thus, the results of an FMEA should feed back into the donor screening and manufacturing processes to minimize risk appropriately.

### Probability of occurrence

For any adventitious agent, it is crucially important to understand the incidence and prevalence of asymptomatic infection, specifically for the donor population, as it sets the baseline for the rest of the assessment. Incidence is a measure of how frequently a new infection occurs in the general donor population over a specific timeframe, and prevalence is a measure of how common the infectious agent is in the donor populace. It is important to monitor these trends which can change gradually or rapidly over time, especially given the omnipresent concern for emerging pathogens. Zika virus, which is shed in human stool, was a significant concern back in 2016, but there are no longer any cases reported in the continental US.^18^ In contrast, cases of Mpox, which is also shed in human stool, rapidly emerged and peaked at >30,000 cases in August 2022 before the introduction of vaccinations.^19^

Specific aspects of the donor population, such as the age and geographic location of donors, can significantly influence the incidence and prevalence of specific infectious agents. A starting point for understanding which agents should be considered are various epidemiological databases and studies conducted in the relevant locale of the donor population.^20,21^ For example, a donor living on the eastern seaboard will have greater risk of infection with *Borrelia*, while a donor in Wyoming will have a greater risk of West Nile virus, which is often asymptomatic and associated with fecal shedding. If epidemiologic data on asymptomatic infection are unknown, then symptomatic incidence and/or prevalence should be used with caution as asymptomatic infection could be more common than symptomatic infection, depending on the etiologic agent.

It is also important to understand transmissibility in relation to the amount of infectious agent shed in stool as compared to estimates of the infectious dose and the potential amount administered via drug product and delivery route. For example, an organism introduced orally can have a different infectious dose than when administered parenterally or per rectum. These combined estimates lend general guidance on transmissibility of a specific infectious agent to inform the approach to laboratory testing or downstream manufacturing processes.

Risk factors associated with carriage of drug-resistant bacteria, including recent antibiotic exposure, may also help guide donor exclusion. However, it is important to recognize that lack of risk factors does not exclude the possibility of carriage. Due to the serious consequences of infection posed by potential transmission of drug-resistant bacteria, including bacteremia and death,^22^ routine screening for drug-resistant bacteria in donor material is prudent in the absence of downstream manufacturing processes designed to reduce or eliminate levels of drug-resistant bacteria.^23^

### Severity of clinical infection and availability of treatment

An important aspect of assessing an adventitious agent is determining the clinical consequences of active infection. Infectious agents that cause severe morbidity and mortality will have a higher risk rating than self-limited infections or those with no or minimal symptoms. Similarly, the availability of efficacious treatment, as well as the adverse effects of treatment, should be taken into account when performing a FMEA. Many resources exist that describe potential sequelae of infection and available treatments (eg, IDSA guidelines, UpToDate).

### Pathogen detectability

The risk of transmitting an adventitious agent is closely tied to the critical elements of donor screening and appropriate quarantine intervals for donor samples. In the best of circumstances, overt evidence of infection through donor questioning and physical examination may be sufficient to evaluate a donor. However, for many infectious agents, asymptomatic carriage is common, mandating testing with caveats related to the level of assay detection and the window of detection, which varies by pathogen. The limit of detection of a given screening assay needs to be assessed relative to asymptomatic carriage levels and the infectious dose of the pathogen. For example, a screening test with a limit of detection higher than the lower limit of infectivity would generate a higher risk of transmission due to a false negative test.

**Figure 1.**
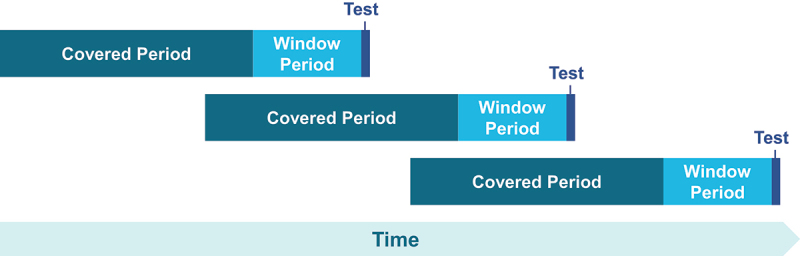
Testing intervals for human donor stool samples must be designed so that subsequent tests cover the window period of the prior test.

A second important factor when examining detectability is how the testing schedule relates to both the “window period” of the pathogen, which can be used to define the quarantine period for the donor sample. The window period is defined as the time from exposure to an infectious agent to first detection of active infection. The duration of the window period will depend on the ramp-up replication time of the infectious agent and the sensitivity of the screening assay. Donations collected during the window period for a specific pathogen should thus be quarantined until a subsequent screening test clears that interval of time, thus determining the “testing interval”. (see Figure 1). Of note, interactions with regulators have indicated a strong desire to quarantine material during this window.

The window period is defined as the time from exposure to an infectious agent to first detection of active infection. The duration of the window period will depend on the ramp-up replication time of the infectious agent and the sensitivity of the screening assay.

### Manufacturing processes and risk mitigations

Donor screening methods always carry the risk of false negatives, whether it be due to test error, a donor being in the window period, inaccurate history from the donor, or deliberate obfuscation on the part of the donor. Furthermore, emerging pathogens can enter the donor pool before they are known to health authorities and before the availability of FDA-cleared laboratory tests to identify infected individuals. SARS-CoV-2 is an example of a recent pandemic where these issues emerged. Even after emergence of the pandemic, recognition of fecal shedding was delayed.^24^ Some donor screening programs were using nasopharyngeal swabs to detect infection although it became evident that fecal shedding can persist for up to 2 months after viral clearance from the respiratory tract.^25^ Once fecal shedding was identified, FDA issued safety alerts regarding potential transmission of SARS-CoV-2 via FMT^4,6^ and outlined that FMT from stool banks donated after December 1st, 2019 should be quarantined until a validated assay for stool matrix was developed, which was not available for several months.^26^

Manufacturing controls can provide an additional level of protection beyond donor screening alone and mitigate risk against transmission of adventitious agents if appropriately designed. First, the manufacturing environment should be controlled and monitored to minimize introduction of other microbial agents into the drug product (ie, contamination). Secondly, adventitious agent inactivation and removal steps should be validated to demonstrate robust removal of adventitious agents under anticipated process conditions.^27,28^ Acceptable validation methods include spiking studies where specific infectious agents of interest vs controls are incorporated into intermediate product materials. Log_10_ reduction factors for the pathogen are quantified over time following the manufacturing processing steps. Washing, which is thought to improve potency and potentially remove undesirable metabolites, is another example of a manufacturing process that has been proposed^29^ that also still requires further formal validation.

Following any inactivation and removal process steps, bioburden or other pathogen testing conducted on the final drug product is an important manufacturing quality control (QC) step to ensure processes were conducted as intended and to assess non-product microbiological contamination. Note, however, that microbial bioburden testing is difficult to conduct for minimally-processed microbiota products that do not undergo adventitious agent inactivation and removal steps.

For an adventitious agents FMEA, the process mitigations for each undesirable organism can be rated against the effectiveness of clearance steps as well as the ability to detect the organism with implemented QC tests on drug product. Higher levels of clearance (typically measured in log_10_-reduction) as well as lower limits of detection (LOD) for downstream identification of residual pathogens, result in lower risk ratings. Without the various drug manufacturing processes detailed above, risk mitigation becomes highly dependent on donor screening and final product testing.

### Example organism FMEA: enteroviruses

Enteroviruses serve as an excellent model pathogen for a donor-stool FMEA. The following discussion includes only non-polio enteroviruses since polio has been largely eradicated in the US through systematic childhood vaccination.

Enterovirus is a genus of positive-sense ssRNA enteric pathogens that typically cause mild self-limited symptoms in a healthy population, such as fever, runny nose, muscle aches, and self-limiting diarrhea.^30^ Rarely, these infections may lead to encephalitis, myocarditis, or acute flaccid paralysis.^30,31^ The incidence of enterovirus in the US is high, ranging from 10 to 15 million infections/year,^32,33^ and enteroviruses are shed in the stool at high levels in asymptomatic patients,^21^ from 4 to 6.5 log_10_/gram.^34^ leading to a “high” probability of occurrence. In contrast, the rating for severity (virulence) is “low”: with the eradication of polio, paralytic infection from poliovirus is virtually unheard of, although vaccine-associated paralytic poliomyelitis is still a possibility.^35^ Non-polio enteroviruses typically cause mild symptoms in a healthy population, such as fever, runny nose, muscle aches, and self-limiting diarrhea,^30^ while rare, more severe disease can lead to encephalitis, myocarditis, or acute flaccid paralysis.^30,31^ Disease course is usually manageable with supportive care with full resolution of symptoms. Despite the generally low severity of clinical illness with enteroviruses, a high potential for recipient exposure to enterovirus from an asymptomatic donor would likely warrant some form of additional controls to prevent transmission. For example, additional screening strategies may mitigate transmission risk, although in the case of enterovirus, no stool-based FDA-cleared test exists. Alternatively, manufacturing methods that selectively reduce adventitious agents could reduce the risk of transmission and replace the need for screening. For example, enteroviruses are small enough that they could be differentially filtered from bacteria, and they have some level of susceptibility to ethanolic inactivation.^36^ Thus, the aggregate results of FMEA applied to enterovirus suggest the need for additional controls which may be achieved through novel screening methods or manufacturing processes to appropriately mitigate risk of transmission.

### Continual adventitious agents surveillance and assessment

**Figure 2.**
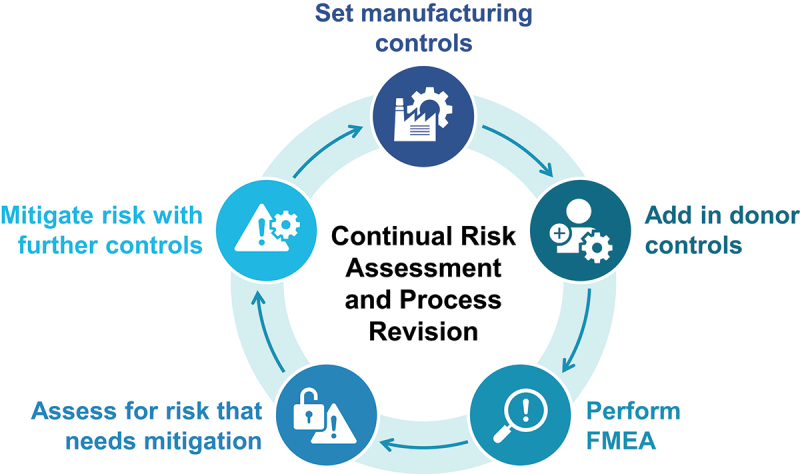
Continual risk assessment and process revision.

Emerging pathogens, evolving clinical and scientific understanding, and development of testing technologies requires ongoing assessment and reevaluation of the threat of adventitious agents for any donor stool program (see Figure 2). Thus, the FMEA exercise should be performed on a regular, scheduled cadence. For emerging pathogens, it is critical to subscribe to early warning alert systems for new clinical syndromes or emerging infections (such as that provided by the Centers for Disease Control and Prevention) and perform an organism-specific FMEA as needed. For known organisms, scheduled risk reassessments are warranted on a periodic basis to assess any new data, particularly on incidence, infectious dose, routes of transmission and novel screening assays.

## Chronic medical conditions

With the exponential growth of microbiome sciences, there has been a growing number of reports suggesting associations between various diseases and the host’s GI microbiome.^37^ The question of whether the transfer of stool from one person to another can transmit a chronic medical condition is important to consider in donor stool risk management. However, establishing causation requires evidence from prospective longitudinal human cohort studies which are expensive, and depending on the disease, may take years to complete.^38^ As a result, germ-free or antibiotic-depleted conventional mice have often been used to determine whether a specific microbe or a microbial community causes a disease.^39^ Human studies are also challenging because a large number of subjects are often needed to account for microbiome variation between individuals and within individuals over time.^40^

An additional layer of complication is the fact that many patients with potential microbiome-associated diseases are treated with medications, which may also directly or indirectly interact with the microbiome.^41^ As a result, the question of whether transplant stool from a person with a specific medical condition can transmit the medical condition to another individual is unsettled for most conditions. Until more is known, it is prudent to exclude donors with most chronic medical conditions, particularly those requiring ongoing medical therapy.

Thus, when evaluating which medical conditions should be permissible or disqualifying in the donor population, an FMEA risk approach, similar to that described for adventitious agents, is appropriate. Important factors to consider are as follows: (1) transmissibility by a stool-derived product; (2) medical consequences of transmission of the disease; (3) the status/persistence of the condition in the donor; and (4) the presence of the associated microbe (or other stool component) in the drug product.

### Transmissibility by a stool-derived product

Given the difficulties in establishing causation between the microbiome and various diseases in the human host, one must have a rigorous approach to assessing the risk of transmissibility. The highest risk scores should be assigned to conditions for which either stool-based or cultivated consortia are being used clinically to improve symptoms and restore the microbiome (either as investigational drugs or approved drugs). Lower risk scores should be assigned to conditions for which there is only one association/transmission study in a rodent model and for which no mechanism of action is provided.

For example, both major depressive disorder (MDD),^42–46^ and generalized anxiety disorder (GAD),^47–50^ have been linked to the gastrointestinal microbiome, and FMT (and/or other LBPs) is being actively pursued as a potential therapeutic. Thus, in this example, these conditions have a high (maximal) score for transmissibility risk in an FMEA.

Another consideration when determining transmissibility risk is if a disease or syndrome may be linked to a specific bacterial species vs a broad range of organisms. For example, prehypertension has been associated with abundance of *Prevotella*, a species that does not form spores.^51–53^ Therefore, in a spore-only based product, the transmissibility risk for prehypertension would be low whereas, in a whole-stool product, transmissibility risk would be higher. These considerations should be taken into account when determining acceptable blood pressure limits for donors. However, given the general lack of established causality for transmission of microbiome-associated chronic medical conditions, the risks of transmission need to be carefully weighened against the benefits of a boader donor pool and general accessibility of donor stool, as described below.

### Medical consequences of transmission of a microbiome-associated disease

As noted above, there are marked limitations in our current knowledge of which chronic medical diseases are associated with general microbiome disruption or specific microbes. When considering the risk of transmitting a disease phenotype, it is important to consider the morbidity and mortality associated with the disease and the availability of efficacious therapy with a favorable safety profile. This type of risk-based approach is the cornerstone of all drug safety evaluations, including donor-derived microbiome therapeutics.

Thus, there may be a higher risk tolerance to permit stool donors with a history of obesity for a product developed to treat an otherwise terminal cancer since the harm from terminal cancer outweighs the potential harm from obesity. In contrast, there would be low risk tolerance to consider obese donors for a product designed to modulate blood sugar, as the risk profile overlaps with the therapeutic goal of the investigational drug.

### Persistence of the condition in donors

Some chronic medical conditions, such as obesity in an otherwise healthy individual, can resolve over time with dietary changes, exercise and other lifestyle modifications. In contrast, some chronic medical conditions are expected to persist. For example, major depressive disorder is viewed as a chronic illness,^54–56^ even when responsive to antidepressant medications, and has been associated with a persistent dysbiotic microbiome signature.^42,57,58^ Thus, when weighing past medical history relative to donor eligibility, the nature and chronicity of any medical illness needs to be carefully assessed until microbiome profiles are better characterized and understood.

### Creating and maintaining a donor screening program

With FMEA rubrics in hand, one can construct a suitable donor screening program incorporating elements described above, along with stringent donor qualification criteria and planned cadences of screening activities.

FMEAs should be used as a tool to assess if there is residual risk that should be mitigated. This is done by setting risk score thresholds, that if exceeded, may require further donor or manufacturing controls. For example, if there is a score of “high” for infectivity, “high” for severity of disease, and “moderate” for pathogen detectability, a pre-defined rubric could flag that combination for further assessment. Mitigation opportunities may include detection assay improvements or development of an inactivation process. Such a rubric can operate with qualitative and/or quantitative descriptors (high, medium, low, etc) with the goal of setting a threshold requiring further action. Sometimes, the risk may be determined to be nonmitigatable, but the residual risk analysis should be documented. Furthermore, because understanding of clinical science, microbiome, and adventitious agents continue to advance, the FMEAs should be reviewed on a periodic basis. This should be coupled with a process for monitoring emerging infectious diseases with a plan to deploy rapid risk assessments, as needed.

Risk mitigation is optimized when thorough and thoughtful donor screening is combined with robust manufacturing processes, which may serve as a safety net if screening fails due to the inherent limitations in laboratory assay performance. When pursuing overlapping donor and manufacturing controls, it is important to weigh the benefits of additional risk mitigation against the added costs and burden of donor screening, which have been well described for FMT.^59^ For example, omission of laboratory screening for drug-resistant bacteria in donor stool prior to initiating manufacturing may be justified by the manufacturing processes coupled with bioburden testing for vegetative bacteria as part of drug product release testing. These decisions are governed by the nature of the product and the manufacturing processes associated with the production of that product. Thus, something that is exceedingly well controlled for at the manufacturing stage (eg, absence of *Staphylococcus aureus* controlled by solvent inactivation and release testing) might not require upstream controls (such as testing for methicillin resistance genes found in methicillin-resistance *S. aureus* in all donor stool).

## Proposed actions for the FMT and donor-derived therapeutics field

This commentary lays out a risk-based approach for strengthening the safety profile of donor-derived microbiome therapuetics (including FMT). Applying and justifying such an approach is standard practice as part of the regulatory approval process for a pharmaceutical, but can also be applied in a targeted way to improve FMT and human-microbiome-derived therapeutics that have not gone through full regulatory approval. We propose the following actions to be adopted by the field, based on our experience with this approach:

- Bracketed testing to control for window periods should be routine in the absence of compensatory manufacturing controls. Almost all infectious disease tests have a window period, which means that a donor could test negative and still be infectious. Most window periods with modern nucleic acid testing commonly used in screening are no more than 10 days, so we recommend that all donors be tested on the day of collection and then 14 days later, before releasing the material that was initially collected.
- Routine freeze-thaw of stool prior to use. Numerous parasites do not have commericially available tests. Parasites, unlike many bacteria, can be susceptible to freeze-thraw^60–62^ and there are not readily available screening assays for most stool pathogens, preventing laboratory testing from being a principal screening approach. The addition of a freeze-thaw step (at least 24 h at ≤-65°C) would help mitigate the risk of parasite transmission.
- Standardized screening for MDROs: Multi-drug resistant organisms represent the largest screening gap for stool-derived therapies, as there are no cleared tests for MDROs (everything available exists in the lab-developed test space) and there are no agreed upon thresholds for concern for transmission. The FDA has previously issued a brief^63^ in 2019 after multiple infections and one patient death and studies have found a prevalence of up to 25% in various donor populations.^64,65^ Stakeholders should come together to devise standards for MDRO screening, including specific tests with described LODs and thresholds for transmission concern. These tests should then be cleared with regulators under clinical regulatory mechanisms (Clinical Laboratory Information Ammendent, i.e. CLIA) with full regulatory review (such as the FDA in the United States) so they can be used across drug licenses, instead of as LDT (lab developed test) or GMP tests utilizable only within a specific drug license.
- Inclusion of SARS-CoV-2 stool screening in the absence of manufacturing controls: As described above, SARS-CoV-2 can persist in stool in the absence of nasal swab NAAT positivity.^25^ Stakeholders should come together to establish a standardized screening protocol with an established LOD, and ideally an industry stakeholder would get this test formally cleared under the appropriate regulatory agency for their location.
- Establish the screening sensitivity/specificity and LOD for the commonly used GI pathogen panels. GI pathogen screening is often done with multi-plexed NAAT tests, such as the Biofire® GI Pathogen panel. These tests are designed to be performed on patients with symptoms to diagnose a specific pathogenic cause of their symptoms, and are not screening assays, although they are used that way in donor screening. As a result, they were designed with a higher pretest probability of infection, and their performance characteristics in terms of sensitivity and specificity in asymptomatic donors may be different than what is described in the package insert. This makes the risk assessments using the available package insert numbers an estimate at best. As a result, we recommend that stakeholders work with these testing manufacturers to establish, and get regulator approval of, the true sensitivity and specificity of these assays when used for screening purposes (ie in asymptomatic individuals under a repeat-testing paradigm).

## Conclusions

Donor-derived products allow for the development of potent therapeutics that cannot currently be derived *ex vivo* or *in vitro*. However, donor-derived products carry intrinsic risks of infectious and chronic disease transmission that must be weighed and mitigated where feasible. A robust and well-reasoned donor program that incorporates medical history, physical examination, laboratory testing, and donor sample inspection are only the first steps in reducing the risk of transmission of infectious agents and chronic conditions. Manufacturing controls are the cornerstone of risk mitigation when screening unwittingly fails. FMEAs can be used as a tool to assess if there is residual risk that should be mediated. This evaluation can lead to further donor or manufacturing controls. Together, a well-reasoned donor program and manufacturing controls are complementary strategies that must be revisited and reexamined frequently with constant vigilance to mitigate risk to patients.

## Data Availability

Data sharing is not applicable to this article as no new data were created or analyzed in this study.
